# Adjuvant Radiotherapy in the Oldest-Old: A National Spanish Multicenter Analysis of Endometrial Cancer Patients Aged ≥ 80 Years

**DOI:** 10.3390/jpm16100520

**Published:** 2026-10-06

**Authors:** Ángeles Rovirosa, Pedro Muñoz, Elisabeth González del Portillo, Sonia García Cabezas, Belén De Paula, Elena García, Diana Gevara Barrera, María Carmen Sánchez Buzón, Joel Mases, Piedad Galdós, Paola Navarrete, Ana Laura Rivero, Pedro J. Prada, Ana De Juan, Jesús Mazaira, Arancha Ayete Andreu, Arantxa Campos, María Cerrolaza, Cristina García, Eugenia Ortega, Dolores Peña, Pilar Samper, Teresa Muñoz, José Domínguez Rullán, Dina Najjari, Andrea Slocker, Javier Anchuelo

**Affiliations:** 1 Hospital Clinic, 08036 Barcelona, Spain; rovirosa@clinic.cat (Á.R.); mases@clinic.cat (J.M.); 2 Gerencia de Atención Primaria Servicio Cántabro de Salud, 39001 Santander, Spain; pedro.munoz@scsalud.es; 3 Instituto de Investigación Valdecilla (IDIVAL), 39011 Santander, Spain; pjprada@pjprada.com (P.J.P.);; 4 Hospital Universitario Rey Juan Carlos, 28935 Móstoles, Spain; gonzalez_beth@hotmail.com (E.G.d.P.); pisamot@gmail.com (P.S.); 5 Hospital Universitario Reina Sofía, 14004 Córdoba, Spain; songar1@gmail.com; 6 Onkologikoa, 20014 Donostia, Spain; 7 Hospital Universitario Nuestra Señora de Candelaria, 38010 Tenerife, Spain; adassamare@gmail.com; 8 Clínica Benidorm, 03500 Alicante, Spain; 9 Hospital Universitario Puerta del Mar, 11011 Cádiz, Spain; 10 Hospital Universitario Marqués de Valdecilla, 39008 Santander, Spain; 11 Hospital General de Sierrallana, 39300 Torrelavega, Spain; 12 Hospital Universitario Miguel Servet, 50009 Zaragoza, Spainacampobo@salud.aragon.es (A.C.); meortegai@salud.aragon.es (E.O.); 13 Hospital Universitario Ramón y Cajal, 28034 Madrid, Spain; tere.miguelanez@gmail.com (T.M.);; 14 Instituto Catalán de Oncología, 08907 Barcelona, Spain; dnajjari@iconcologia.net (D.N.);

**Keywords:** endometrial cancer, adjuvant radiotherapy, older adults, personalized medicine, geriatric oncology

## Abstract

**Objectives**: The incidence of endometrial cancer is rising among elderly women due to population aging. However, patients aged ≥ 80 years remain underrepresented in clinical trials, and their management is often influenced by age rather than clinical suitability. This multicenter retrospective study aimed to evaluate treatment patterns, oncologic outcomes, prognostic factors, and the safety of adjuvant radiotherapy (RT) in Spanish women aged ≥ 80 years with surgically treated endometrial cancer. **Methods**: We analyzed data from 353 patients aged ≥ 80 years treated with curative-intent surgery between 2003 and 2019 across 11 centers. Adjuvant RT included external beam radiotherapy (EBRT), vaginal brachytherapy (VB), or both. Toxicity was graded using CTCAE v5.0. Survival outcomes were assessed using Kaplan–Meier and Cox regression analyses. **Results**: The median age was 83 years; 83.5% had early-stage disease. Adjuvant RT was administered to 227 patients (64.3%), and chemotherapy to only 13. Grade ≥ 3 toxicity was rare (<1%). Five-year outcomes were as follows: local control (88.9%), cancer-specific survival (68.7%), and overall survival (58.7%). In multivariable analysis, VB alone and combined EBRT + VB were independently associated with improved local control. No RT modality was independently associated with overall survival, although favorable trends were observed for VB alone and EBRT + VB. Higher comorbidity burden (CCI ≥ 5), but not age ≥ 85 years, was independently associated with worse overall survival. **Conclusions**: Adjuvant RT was associated with favorable oncologic outcomes and a low incidence of severe toxicity in women aged ≥ 80 years with endometrial cancer. These findings support individualized treatment based on tumor characteristics and comorbidity rather than chronological age alone. A multidisciplinary approach incorporating geriatric assessment may further support personalized treatment decision-making in this growing patient population.

## 1. Introduction

Endometrial cancer is the most common gynecologic cancer in developed countries, and its incidence increases with age, with a mean age at diagnosis of approximately 68 years [1]. It is estimated that in the next 20 years, there will be a significant increase in the number of patients over 65 years diagnosed with cancer, including endometrial cancer, due to population aging and obesity [2]. However, older adults remain underrepresented in clinical trials, leaving a gap in the evidence available to guide the optimal management of this population [1,3,4].

Older patients often present with tumors with more aggressive characteristics and at more advanced stages at diagnosis compared with younger patients, which contributes to a worse prognosis [5]. In addition, these patients receive fewer adjuvant treatments, such as radiotherapy and chemotherapy, which may also contribute to poorer oncologic outcomes [1,6]. Accurate diagnostic assessment is therefore particularly relevant in postmenopausal women. Transvaginal ultrasonography remains an important first-line imaging tool, and parameters such as endometrial thickness may help identify women at increased risk of endometrial malignancy. In addition, uterine artery Doppler indices have been investigated as complementary sonographic markers for differentiating benign from malignant endometrial changes in women with postmenopausal bleeding [7].

The molecular classification of endometrial cancer has substantially changed our understanding of the disease by dividing it into four distinct subgroups. These subgroups not only reflect differences in tumor biology but also have significant clinical implications for prognosis and treatment response. However, clinical guidelines differ in their recommendations regarding the incorporation of molecular classification into routine clinical practice. Guidelines from the European Society for Radiotherapy and Oncology (ESTRO), the European Society for Medical Oncology (ESMO), and the American Society for Radiation Oncology (ASTRO) differ in the extent to which molecular features are incorporated into treatment recommendations, with ASTRO guidelines adopting a more conservative approach [8]. Moreover, it remains unclear how to integrate classical factors, such as lymphovascular space invasion (LVSI) or age, with the molecular classification, which adds another layer of complexity to clinical decision-making [8,9,10]. More recent European guidelines have further refined the integration of molecular and traditional clinicopathologic factors into risk stratification and treatment decision-making [11].

In recent years, a decrease in survival has been observed among patients diagnosed with endometrial cancer. This trend may partly reflect the increasing proportion of older patients, who frequently present with greater comorbidity and may receive less intensive treatment [12]. As the population ages, it is increasingly necessary to better understand how advanced age influences treatment selection and oncologic outcomes. However, representation of patients aged ≥ 80 years in key studies, such as the Endometrial Cancer Atlas, has been scarce, limiting the applicability of these findings to this age group [13].

In this context, the PORTEC-4A study and the RAINBO program have been designed to evaluate molecularly informed treatment strategies in endometrial cancer [14,15]. PORTEC-4A evaluates the use of an integrated molecular risk profile to guide adjuvant treatment decisions, while the RAINBO investigates molecularly directed adjuvant treatment strategies across the different molecular subgroups of endometrial cancer. These studies are expected to further refine risk stratification and support more personalized treatment approaches. However, evidence specifically addressing patients aged ≥ 80 years remains limited, underscoring the need for real-world data in this population.

## 2. Materials and Methods

This was a retrospective, multicenter cohort study of patients aged ≥ 80 years who underwent primary surgical treatment for endometrial cancer in 11 Spanish hospitals between 2003 and 2019. Data were extracted from electronic medical records after approval was obtained from all local institutional ethics committees (code number: 2019.164). Clinical information was collected using standardized electronic forms and transferred to a central coordinating hospital, where all data were stored and processed.

Eligible patients were women aged ≥ 80 years with a histopathological diagnosis of endometrial cancer who underwent surgery with curative intent as their initial treatment. Patients with distant metastases at diagnosis or treated with palliative intent were excluded. Patients with missing data required for a specific outcome analysis were excluded only from the corresponding analysis but were retained for descriptive analyses and other outcomes when data were available.

The initial diagnosis of endometrial cancer was histologically confirmed using endometrial tissue sampling in all patients. Definitive histological subtype, tumor grade, and other pathological characteristics were established from the hysterectomy specimen. Recurrent disease was diagnosed based on clinical and/or radiological findings and was histologically confirmed by biopsy whenever clinically feasible.

**Figure 1:**
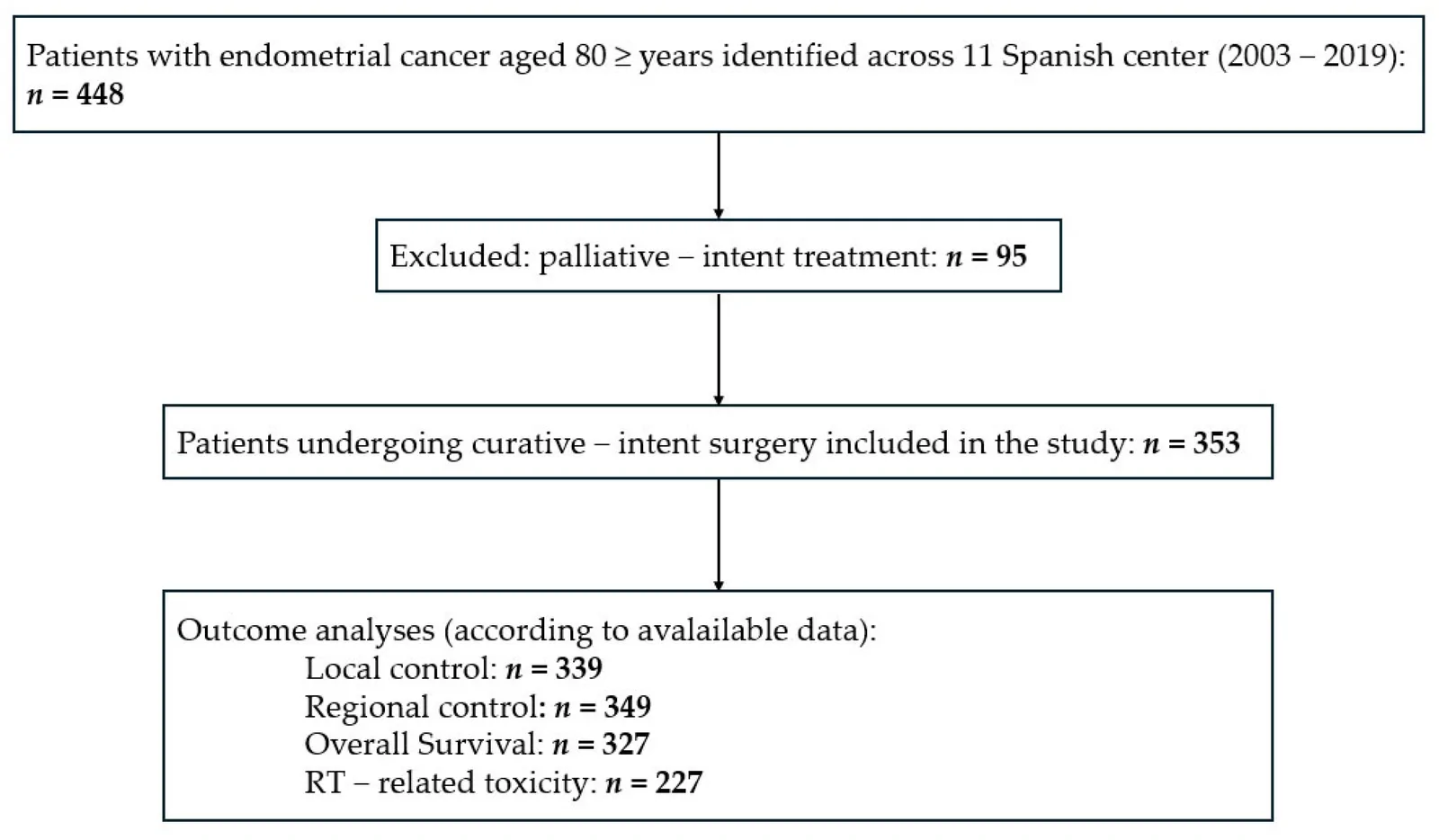
Flowchart of patient selection.

The study cohort included 353 patients who underwent surgery with curative intent. Because of missing outcome-specific data, the number of patients included in each analysis varied according to the endpoint: 327 patients were included in the analysis of overall survival, 320 in cancer-specific survival, 339 in local control and 349 in regional recurrence. For radiotherapy-related toxicity analysis, the 227 patients who received adjuvant radiotherapy were evaluated (Figure 1).

The following variables were recorded:

- Demographics: age (categorized as 80–84 vs. ≥85 years) and Charlson Comorbidity Index (CCI), categorized as <5 vs. ≥5.
- Tumor characteristics: histological subtype (endometrioid, serous, clear cell, carcinosarcoma, dedifferentiated, other or unknown), tumor grade (G1, G2, G3, or unknown), FIGO stage (2009 classification), lymphovascular space invasion (LVSI), and peritoneal cytology.
- Preoperative imaging: transvaginal ultrasound, computed tomography (CT), magnetic resonance imaging (MRI), and positron emission tomography/computed tomography (PET/CT).
- Surgical details: surgical approach (laparotomy, laparoscopy, vaginal), type of hysterectomy, lymphadenectomy (pelvic and/or para-aortic), and sentinel lymph node biopsy.
- Adjuvant therapy: external beam radiotherapy (EBRT), vaginal brachytherapy (VB), or a combination of both, chemotherapy administration, total dose, number of fractions, radiotherapy technique (2D, 3D-conformal radiotherapy, volumetric modulated arc therapy, tomotherapy), and boost administration.
- Toxicity: acute and late toxicity by organ system (vaginal, urinary, rectal, intestinal).
- Follow-up data: site and timing of recurrence (vaginal, nodal, distant), survival status, and time to event (months).

All patients underwent surgery with curative intent. Postoperative management was determined according to the clinicopathological risk factors available at the time of treatment and institutional practice, including FIGO stage, histological subtype, tumor grade, lymphovascular space invasion, and nodal status when available.

Adjuvant radiotherapy consisted of vaginal brachytherapy (VB) alone, external beam radiotherapy (EBRT) alone, or combined EBRT and VB. The indication and modality of adjuvant radiotherapy were individualized according to clinicopathological recurrence risk, patient comorbidities and overall clinical condition, institutional practice, and the treatment guidelines applicable during the study period. Given the retrospective multicenter design and the long study period (2003–2019), treatment decisions were not based on a single uniform protocol across participating centers.

External beam radiotherapy was delivered with total doses ranging from 45 to 50.4 Gy in 25–28 fractions of 1.8–2 Gy, five days per week. A boost was administered in selected cases with residual disease or positive surgical margins.

Vaginal brachytherapy was performed using high-dose-rate iridium-based brachytherapy or electronic brachytherapy. Fractionation schedules varied between institutions depending on whether VB was used as stand-alone adjuvant treatment or as a boost following EBRT.

Due to the low frequency of grade ≥ 3 events, toxicity was analyzed as a binary variable (any toxicity vs. no toxicity) in multivariable analyses.

Lymphovascular space invasion (LVSI) was defined as the presence of tumor cells within endothelial-lined lymphatic or vascular spaces on histopathological examination and was categorized as present or absent according to the pathology report.

Histological subtype was classified according to the final surgical pathology report as endometrioid, serous, clear cell, carcinosarcoma, dedifferentiated, or unknown. Endometrioid histology was used as the reference category in the regression analyses.

Tumor grade was obtained from the final surgical pathology report and categorized as grade 1 (G1), grade 2 (G2), grade 3 (G3), or unknown when tumor grade was not available.

Disease stage was classified according to the 2009 FIGO staging system and was categorized as early-stage (FIGO I–II) or advanced-stage disease (FIGO III–IV) for regression analyses.

Adjuvant radiotherapy was categorized according to the treatment received as external beam radiotherapy (EBRT) alone, vaginal brachytherapy (VB) alone, combined EBRT and VB, or no radiotherapy (reference category).

Radiotherapy-related complications were retrospectively assessed and graded according to the Common Terminology Criteria for Adverse Events, version 5.0 (CTCAE v5.0) [16]. Toxicities occurring within 90 days after completion of radiotherapy were classified as acute, whereas those occurring more than 90 days after completion of radiotherapy were considered late toxicities. Rectal, urinary, vaginal, and intestinal adverse events were recorded.

Comorbidity burden was assessed using the Charlson Comorbidity Index (CCI), calculated according to the standard Charlson scoring system based on the presence and weighting of predefined comorbid conditions. The original, non-age-adjusted CCI was used; therefore, chronological age was not incorporated into the CCI score and was evaluated separately as an independent prognostic variable. For statistical analyses, the CCI was categorized as <5 or ≥5, based on previously described CCI categories (0, 1–2, 3–4, and ≥5), with scores ≥ 5 representing the highest comorbidity category [17].

Local control (LC) was defined as the time from surgery to the first vaginal recurrence. Regional control (RC) was defined as the time from surgery to the first pelvic and/or para-aortic nodal recurrence. For each control endpoint, patients without the corresponding event were censored at the date of last follow-up.

Disease-free survival (DFS) was defined as the time from surgery to the first documented recurrence (local, regional, or distant), with patients without recurrence censored at the date of last follow-up. Overall survival (OS) was defined as the time from surgery to death from any cause, with patients alive at the end of follow-up censored at the date of last follow-up. Cancer-specific survival (CSS) was defined as the time from surgery to death attributable to endometrial cancer. Only deaths attributable to endometrial cancer were considered events; deaths from other causes were censored at the date of death, and patients alive at the end of follow-up were censored at the date of last follow-up.

Categorical variables were described using absolute numbers and percentages; Pearson’s chi-square test was used to compare the treatment groups (no radiotherapy vs. radiotherapy). Time-to-event outcomes were analyzed using the Kaplan–Meier method for local control, regional control, cancer-specific survival, and overall survival. Differences between groups were assessed using the log-rank test. Prognostic factors associated with LC, DFS, and OS were evaluated using Cox proportional hazards regression models.

Before fitting the multivariable Cox proportional hazards regression model, multicollinearity among the independent variables was thoroughly assessed using the variance inflation factor (VIF) and tolerance statistics derived from linear regression diagnostics. Variables with a VIF greater than 5 or a tolerance lower than 0.20 were flagged for potential multicollinearity. Additionally, the proportional hazards assumption for continuous and categorical predictors was evaluated to ensure model validity.

To account for selection bias and confounding by indication inherent in the observational design, an inverse probability of treatment weighting (IPTW) approach was utilized [18,19]. First, a propensity score for each patient was estimated using a multivariable logistic regression model including the following baseline covariates: age, Charlson Comorbidity Index, histological subtype, tumor grade, type of surgery, FIGO stage and lymphovascular space invasion. Stabilized weights were calculated to preserve the original sample size and mitigate the influence of extreme weights [20,21,22].

Variables with a *p*-value < 0.20 in the univariable analysis were included in the multivariable analysis, following the criterion proposed by Hosmer and Lemeshow [23] and shared by other reference authors [24]. The tables present the HR from the univariable analysis and the adjusted HR (aHR) for the variables included in the multivariable analysis.

To estimate the final treatment effect on survival, a Cox proportional hazards regression model was applied to the weighted pseudopopulation. Because IPTW alters the sample structure, bootstrapping with 1000 resamples was performed to obtain robust standard errors, *p*-values, and 95% confidence intervals (95% CI) for the estimated hazard ratio (HR).

## 3. Results

### 3.1. Patients and Preoperative Work-Up

Between January 2003 and December 2019, a total of 353 patients aged ≥ 80 years were diagnosed with endometrial cancer and underwent surgery with curative intent. The median age of the cohort was 83 years (range, 80–95). A total of 250 patients (70.8%) were aged 80–84 years, while 103 patients (29.2%) were aged ≥ 85 years. These two age groups were used for subsequent analyses. The median follow-up was 41.3 months (range, 1–196).

The median Charlson Comorbidity Index (CCI) was 6 (range, 2–15). The most common histological subtype was endometrioid adenocarcinoma (242 patients, 68.6%), followed by serous carcinoma (66 patients, 18.7%), clear cell carcinoma (16 patients, 4.5%), carcinosarcoma (17 patients, 4.8%), and dedifferentiated carcinoma (2 patients, 0.6%). Other histological subtypes were identified in 7 patients (2.0%), while the histological subtype was unknown in 3 patients (0.8%). Low-grade tumors (G1) accounted for 94 patients (26.6%), intermediate-grade tumors (G2) for 117 patients (33.1%), and high-grade tumors (G3) for 136 patients (38.5%). Tumor grade was unknown in six patients (1.7%).

Preoperative imaging included transvaginal ultrasound in 299 patients (84.7%), computed tomography (CT) in 186 patients (52.7%), magnetic resonance imaging (MRI) in 132 patients (37.4%), and positron emission tomography/computed tomography in 16 patients (4.5%).

### 3.2. Surgical Treatment and Pathological Findings

A total of 220 patients (62.3%) underwent total hysterectomy with bilateral salpingo-oophorectomy. Sentinel lymph node biopsy was performed in five patients (1.4%), pelvic lymphadenectomy in 72 patients (20.4%), combined pelvic and para-aortic lymphadenectomy in 33 patients (9.4%), and extended lymphadenectomy with omentectomy and appendectomy in nine patients (2.5%).

Regarding surgical approach, laparoscopy was performed in 264 patients (74.8%), laparotomy in 69 patients (19.5%), and vaginal surgery in 11 patients (3.1%).

**Figure 2:**
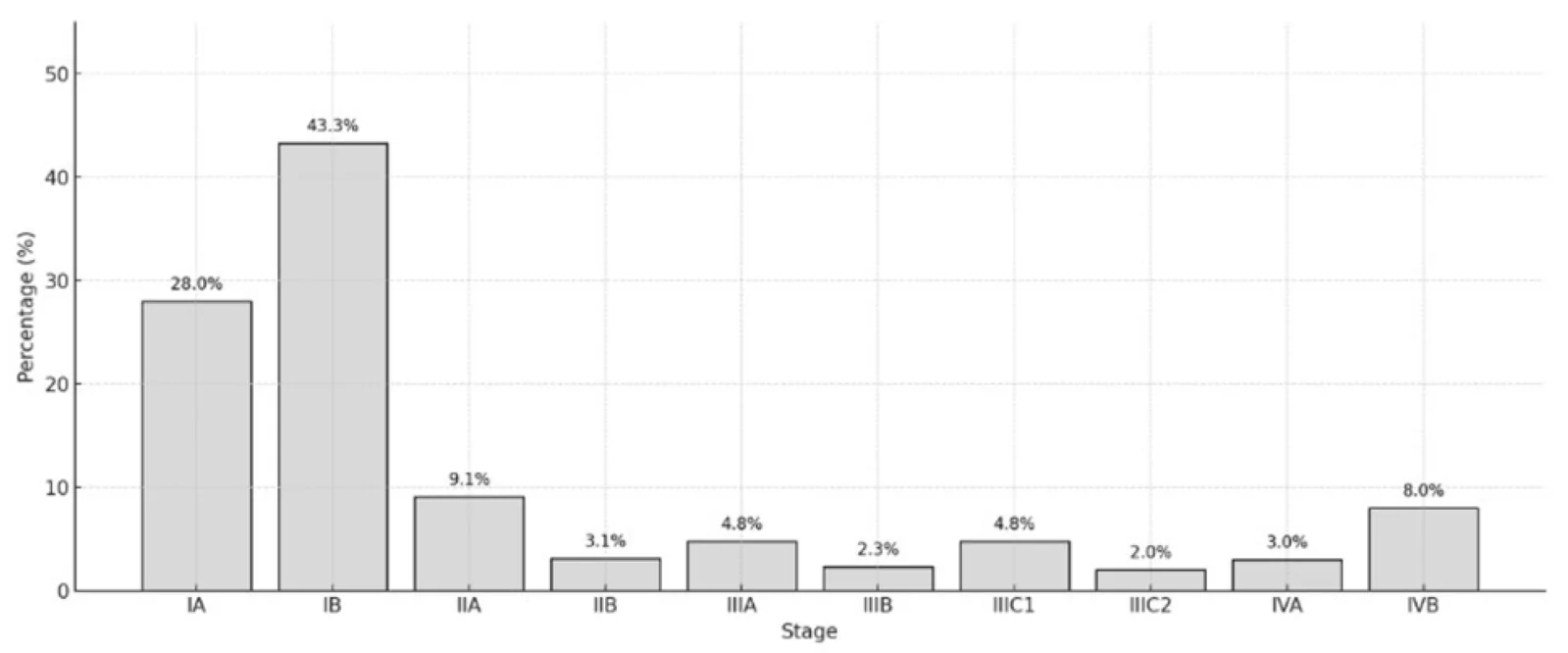
Pathological FIGO 2009 stage distribution.

Most patients (295 patients, 83.6%) presented with early-stage disease (FIGO stages I–II), whereas 53 patients (15.0%) had FIGO stage III–IV disease, and the stage was unknown in 5 patients (1.4%) (Figure 2). Lymphovascular space invasion was present in 113 patients (32%). Node-negative disease was confirmed in 109 patients (30.9%). Positive pelvic lymph nodes were identified in 15 patients (4.2%), combined pelvic and para-aortic nodal involvement in 15 patients (4.2%), and isolated para-aortic metastases in 3 patients (0.8%). Peritoneal cytology was performed in 173 patients, of whom 14 (8.1%) had positive cytology. Residual disease after surgery was documented in 18 patients (5.1%).

### 3.3. Adjuvant Treatment

Adjuvant treatment was indicated based on clinicopathologic features and institutional protocols. Overall, 227 patients (64.3%) received adjuvant radiotherapy. Of these, 116 patients (32.9%) received combined external beam radiotherapy (EBRT) and vaginal brachytherapy (VB), 28 patients (7.9%) received EBRT alone, and 83 patients (23.5%) received VB alone.

Regarding EBRT technique, three-dimensional conformal radiotherapy was used in 108 patients (75.0%), volumetric modulated arc therapy in 14 patients (9.7%), tomotherapy in 20 patients (13.9%), and two-dimensional techniques in 2 patients (1.4%). Seventeen patients (11.8%) with residual disease received a boost dose ranging from 10 to 19.8 Gy. Four patients (2.8%) did not complete the planned EBRT course.

For vaginal brachytherapy, 37 patients (18.6%) were treated with electronic brachytherapy, while the remaining 162 patients (81.4%) received iridium-based high-dose-rate brachytherapy. Fractionation data were available for 191 patients. Fractionation schedules varied: 13 patients (6.8%) received one fraction, 43 (22.5%) received two fractions, 44 (23%) received three fractions, 38 (19.9%) received four fractions, 18 (9.4%) received five fractions, and 35 (18.3%) received six fractions. The mean number of fractions was 3.6 (median, 3). Dose per fraction ranged from 4 to 7.5 Gy (mean, 5.1 Gy; median, 5 Gy), depending on institutional protocols and treatment intent.

Adjuvant chemotherapy was administered to 13 patients (3.7%). The most commonly used regimen was carboplatin plus paclitaxel, followed by platinum-based combinations. After disease recurrence, a higher number of patients (20 patients) received systemic chemotherapy, suggesting greater consideration of systemic treatment in the relapse setting than in the adjuvant context.

### 3.4. Toxicity

Radiotherapy was generally well tolerated, with a low incidence of severe acute and late toxicity.

#### 3.4.1. Acute Toxicity

Acute rectal toxicity occurred as grade 1 in 30 patients (13.2%), grade 2 in nine patients (4.0%), and grade 3 in one patient (0.4%). Urinary toxicity was grade 1 in 38 patients (16.7%) and grade 2 in 12 patients (5.3%). Vaginal toxicity was grade 1 in 39 patients (17.2%) and grade 2 in 6 patients (2.6%). Intestinal toxicity was grade 1 in 29 patients (12.8%) and grade 2 in 19 patients (8.4%), with no grade ≥ 3 events.

#### 3.4.2. Late Toxicity

Rectal toxicity (grade 1–2) occurred in 4 patients (1.8%), with one grade 3 event (0.4%). Urinary toxicity (grade 1–2) was observed in 13 patients (5.7%), with no grade ≥ 3 events. Vaginal toxicity (grade 1–2) occurred in 25 patients (11.0%), with two grade 3 events (0.9%). Intestinal toxicity (grade 1–2) was observed in 11 patients (4.8%), with grade 3 toxicity reported in 1 patient (0.4%). No grade 4 or 5 radiotherapy-related toxicities were observed.

Due to the low incidence of grade ≥ 3 events, toxicity was dichotomized into presence versus absence of any toxicity for further analysis. No statistically significant association was found between toxicity and any clinical or treatment-related factors.

### 3.5. Oncologic Outcomes

During follow-up, 35 patients (9.9%) experienced vaginal vault recurrence, 19 patients (5.4%) developed pelvic nodal recurrence, 13 patients (3.7%) had combined pelvic and para-aortic nodal recurrence, and 13 patients (3.7%) presented with isolated para-aortic nodal recurrence. The median time to nodal relapse was 16.9 months (interquartile range, 13.9). Distant metastases occurred in 58 patients, with a median time to distant relapse of 14.5 months (interquartile range, 15.9).

Patients were divided into two groups according to receipt of any form of radiotherapy. Comparative analyses are summarized in Table 1. Patients who received radiotherapy were more frequently aged 80–84 years than those who did not receive radiotherapy (76.7% vs. 60.3%; *p* = 0.001). Patients receiving radiotherapy also had a higher proportion of CCI ≥ 5 (78.8% vs. 70.2%), although this difference did not reach statistical significance (*p* = 0.073). No significant differences were observed between groups regarding histological subtype, tumor grade, lymphovascular space invasion, or FIGO stage.

**Figure 3:**
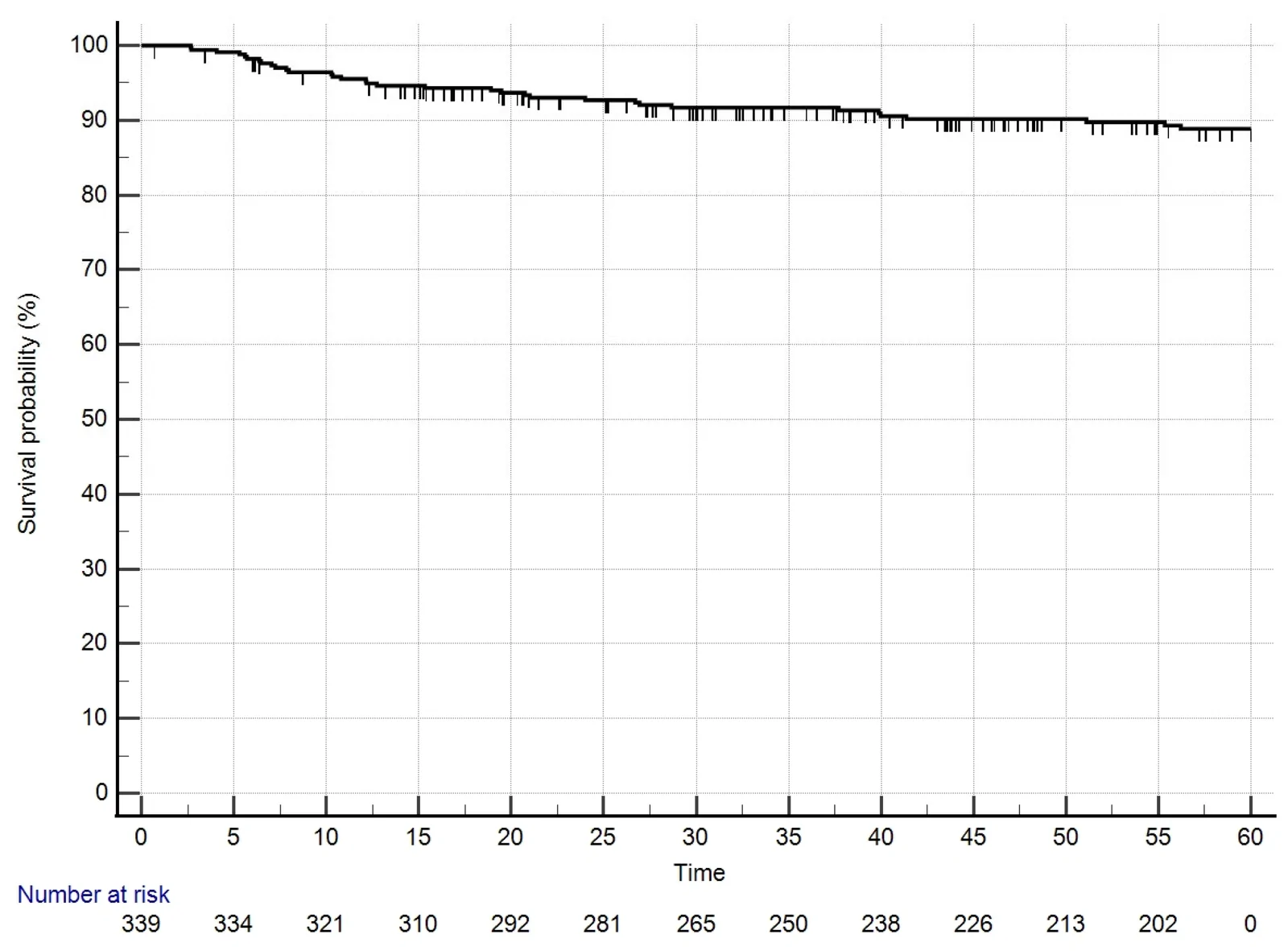
Local control-5 years.

**Figure 4:**
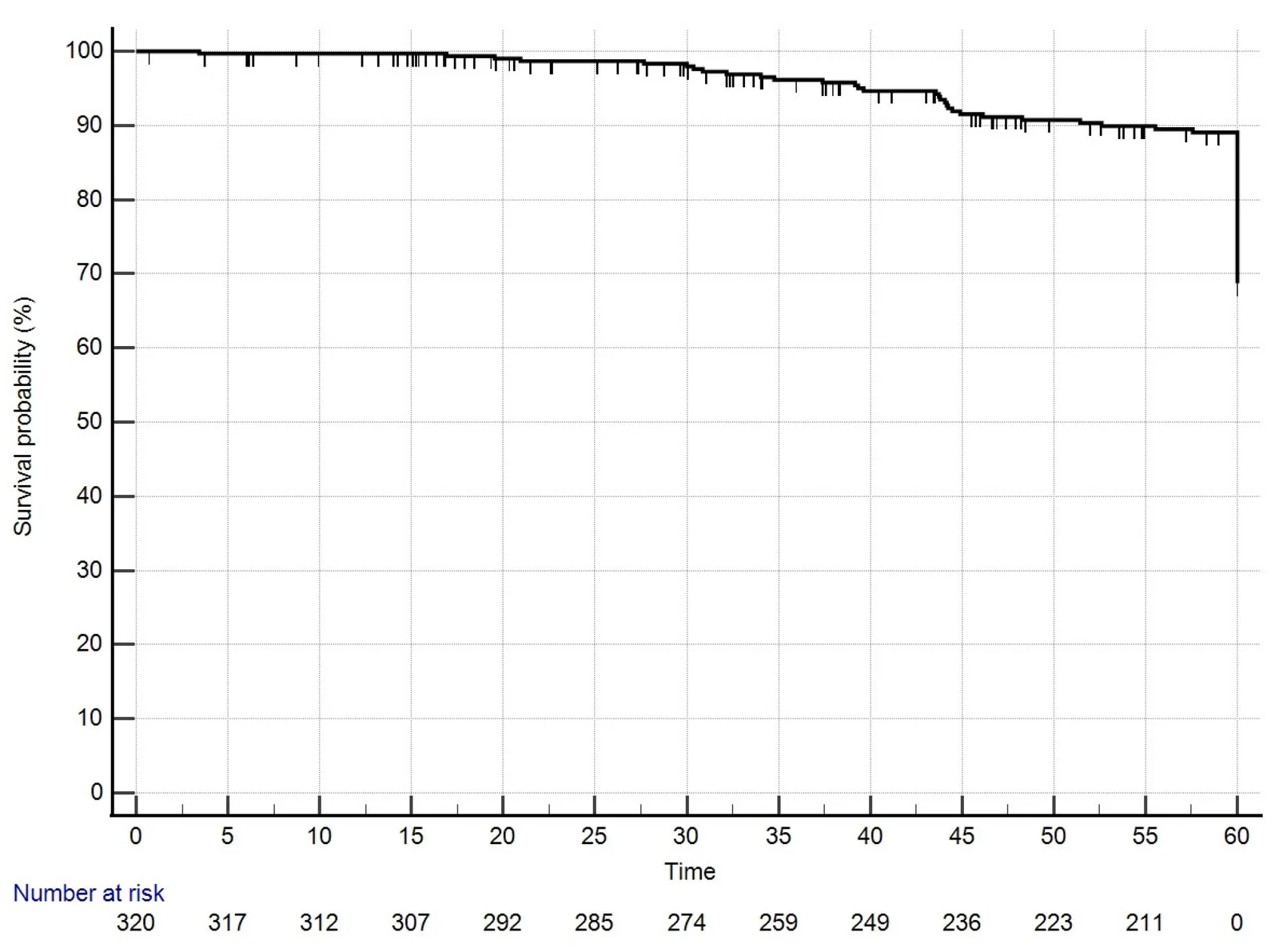
Cancer—specific survival—5 years.

Local control (LC) rates at 1, 2, and 5 years were 95.2%, 92.7%, and 88.9%, respectively (Figure 3). Regional control (RC) rates at 1, 2, and 5 years were 96.6%, 91.4%, and 87.8%, respectively. Cancer-specific survival (CSS) rates at 1, 2, and 5 years were 99.7%, 98.3%, and 68.7%, respectively (Figure 4). Overall survival rates at 1, 2, and 5 years were 90.3%, 73.9%, and 58.7%, respectively.

The balance between the treatment groups (RT vs. no RT) is shown in Supplementary Table S1. Prior to inverse probability of treatment weighting (IPTW) adjustment, substantial imbalances were observed between the study groups, particularly regarding age (80–84 years; SMD = 0.359) and type of surgery (HA + DA; SMD = 0.436).

Following the application of IPTW weights, an optimal balance (defined as a final SMD < 0.10) was successfully achieved for the vast majority of baseline covariates. Specifically, differences in the distribution of age (SMD = 0.002), Charlson Comorbidity Index (SMD = 0.014), tumor grade (SMD = 0.040), and histology (SMD = 0.091) were drastically reduced, ensuring group comparability across these factors. FIGO stage and lymphovascular invasion maintained their initial balanced distribution (SMD = 0.032 and SMD = 0.094, respectively).

However, the type of surgery (HA + DA) exhibited a clinically relevant residual imbalance, showing minimal changes in its proportions and maintaining an elevated post-adjustment SMD (SMD = 0.360). This was one of the three variables not included in the multivariable analysis—along with FIGO stage and lymphovascular invasion—due to failure to meet the established statistical significance threshold of *p* < 0.20 in the univariable analysis.

The IPTW weights ranged from 1.19 to 6.23, with a mean of 2.01 and a median of 1.70, indicating an absence of disproportionate weights.

Supplementary Table S2 presents the HRs, 95% confidence intervals, and *p*-values from the multivariable analysis, including the five variables that met the established selection criterion (*p* < 0.20). It can be observed that, upon adjusting for covariates—thereby making the groups more comparable—the factors independently associated with higher 5-year mortality were a Charlson Comorbidity Index ≥ 5 (aHR 2.36), the serous histological subtype (aHR 2.08), carcinosarcoma (aHR 2.71), and dedifferentiated carcinoma (aHR 7.52). Age ≥ 85 years, tumor grade, and radiotherapy modalities did not show a statistically significant association after weighting.

In the collinearity assessment, all variables showed a tolerance > 0.7 and a VIF < 1.5, indicating that collinearity was weak or nonexistent. In the multivariable analysis for local control, statistically significant factors included: FIGO III–IV vs. I-II (HR 5.37; 95% CI 1.98–14.68; *p* = 0.0011), serous histology (HR 7.22; 95% CI 2.43–21.44; *p* = 0.0004), carcinosarcoma (HR 10.56; 95% CI 2.89–38.57; *p* = 0.001), EBRT + VB (HR 0.069; 95% CI 0.009–0.533; *p* = 0.0108), and VB alone (HR 0.169; 95% CI 0.046–0.558; *p* = 0.0038).

In the multivariable analysis for disease-free survival (DFS), serous histology (HR 4.71; 95% CI 2.58–8.61; *p* < 0.0001), clear cell carcinoma (HR 9.71; 95% CI 3.90–24.17; *p* < 0.0001), carcinosarcoma (HR 7.97; 95% CI 2.91–21.83; *p* = 0.0001), and dedifferentiated histology (HR 8.35; 95% CI 1.08–64.07; *p* = 0.04) were independently associated with a higher risk of disease recurrence. Lymphovascular space invasion (HR 2.22; 95% CI 1.28–3.85; *p* = 0.0045) and unknown tumor grade (HR 5.11; 95% CI 1.49–17.53; *p* = 0.0095) were also independently associated with worse DFS.

For overall survival, the multivariable analysis identified CCI ≥ 5 (aHR 2.36; 95% CI 1.30–4.26; *p* = 0.005), serous histology (aHR 2.08; 95% CI 1.30–3.33; *p* = 0.002), carcinosarcoma (aHR 2.71; 95% CI 1.03–7.13; *p* = 0.043), and dedifferentiated histology (aHR 7.52; 95% CI 4.03–14.03; *p* < 0.001) as independent factors associated with worse overall survival. No radiotherapy modality was independently associated with overall survival in the adjusted model (global *p* = 0.086) (Table 2).

Although patients receiving radiotherapy were younger than those who did not receive radiotherapy (*p* = 0.001), age ≥ 85 years was not independently associated with overall survival after adjustment (aHR 1.25; 95% CI 0.87–1.79; *p* = 0.226). Compared with no radiotherapy, EBRT alone (aHR 1.24; 95% CI 0.69–2.24; *p* = 0.479), VB alone (aHR 0.71; 95% CI 0.49–1.03; *p* = 0.073), and combined EBRT + VB (aHR 0.70; 95% CI 0.47–1.06; *p* = 0.091) were not independently associated with overall survival.

## 4. Discussion

This multicenter retrospective study represents the largest national analysis to date of women aged 80 years or older with endometrial cancer treated with curative-intent surgery, with or without adjuvant radiotherapy. Including 353 patients across 11 centers in Spain, it provides valuable real-world data on oncologic outcomes, prognostic factors, treatment-related toxicity, and patterns of adjuvant treatment in a population that remains underrepresented in prospective trials.

Our results suggest that adjuvant radiotherapy is associated with favorable local disease control and low rates of severe toxicity in elderly women, even beyond the age of 80. The 5-year local control rate of 88.9% is consistent with previous reports showing favorable outcomes in elderly patients treated with radiotherapy [1,6], including a 5-year local control rate of 89.5% reported in a cohort of patients aged ≥ 80 years receiving postoperative radiotherapy [25]. In the multivariable analysis, both EBRT + VB and VB alone were independently associated with a lower risk of local recurrence. Regarding overall survival, no radiotherapy modality reached statistical significance after adjustment for other prognostic factors; however, both VB alone (aHR 0.71; 95% CI 0.49–1.03; *p* = 0.073) and EBRT + VB (aHR 0.70; 95% CI 0.47–1.06; *p* = 0.091) showed non-significant trends toward better overall survival. These findings support the role of adjuvant radiotherapy in optimizing local disease control in appropriately selected elderly patients, although no independent association with overall survival was demonstrated [26].

Several factors associated with worse prognosis were identified, although their impact differed according to the oncologic endpoint evaluated. Advanced FIGO stage, serous histology, and carcinosarcoma were independently associated with worse local control. Disease-free survival was adversely affected by non-endometrioid histologies, particularly serous carcinoma, clear cell carcinoma, carcinosarcoma, and dedifferentiated carcinoma, as well as by lymphovascular space invasion and unknown tumor grade. Regarding overall survival, a high comorbidity burden (CCI ≥ 5), serous histology, carcinosarcoma, and dedifferentiated histology were independently associated with worse outcomes. These findings are consistent with those reported in large retrospective series, including studies from the FRANCOGYN group, which demonstrated that classical prognostic factors retain their predictive value even in older populations [3,27].

A study conducted by the FRANCOGYN group, which included 1228 surgically treated patients across eight French centers, concluded that patients over 70 years of age had tumors with more aggressive histologies and were treated with fewer lymphadenectomies and adjuvant therapies than younger patients, which could partly explain their poorer survival [3]. Similarly, elderly patients with high-risk endometrial cancer underwent lymphadenectomy less frequently and received less chemotherapy and had significantly lower disease-free survival compared with younger patients [26,27]. This pattern of undertreatment persists across age groups, with patients aged ≥ 75 years receiving fewer pelvic and para-aortic lymphadenectomies despite presenting with more advanced disease [26]. Very elderly patients (≥80 years) have been shown to have particularly poor outcomes when systemic treatments are omitted or not administered according to standard treatment recommendations [28]. In our cohort, however, age ≥ 85 years was not independently associated with overall survival after multivariable adjustment, whereas a higher comorbidity burden (CCI ≥ 5) remained an independent adverse prognostic factor. This finding suggests that comorbidity burden may provide more clinically relevant prognostic information than chronological age alone when evaluating very elderly patients for treatment.

Appropriate surgical management also relies on accurate preoperative diagnosis and adequate histopathological evaluation. The importance of accurate pathological assessment before surgical treatment has also been highlighted in the context of gynecologic surgery, particularly when surgical procedures may influence subsequent disease dissemination [29]. Accurate histopathological assessment is also important in the differential diagnosis of uterine lesions, as some rare benign tumors may display unusual morphological or infiltrative growth patterns that can mimic malignancy [30]. In our cohort, all patients had histological confirmation of endometrial cancer by endometrial biopsy before surgery, with definitive pathological characteristics established from the hysterectomy specimen.

Rovirosa et al. analyzed postoperative radiotherapy outcomes across three age cohorts (<65 years, 65–79 years, and ≥80 years). Although late bladder, rectal, and vaginal toxicities were comparable across age groups, the oldest cohort showed higher rates of severe late small bowel toxicity and vaginal cuff recurrences. While cancer-specific survival was lower in patients aged ≥ 80 years, overall survival was similar among the three age groups, supporting the concept that chronological age alone should not preclude consideration of curative treatment approaches, a finding consistent with our multivariable analysis [5,31].

Our results suggest that chronological age alone may not be a limiting factor for curative-intent treatment. Several studies have shown that elderly patients with endometrial cancer are less likely to receive adjuvant radiotherapy despite having clear indications, often due to age-related bias rather than objective assessment of frailty or comorbidity [4,26]. This undertreatment has been associated with higher rates of local recurrence and worse cancer-specific survival. In our cohort, both VB alone and combined EBRT + VB were independently associated with improved local control, while severe radiotherapy-related toxicity was uncommon among patients receiving radiotherapy. Although no radiotherapy modality was independently associated with overall survival, these findings support the use of adjuvant radiotherapy for local disease control in appropriately selected very elderly patients.

Despite a substantial comorbidity burden, reflected by a median Charlson Comorbidity Index of 6, treatment-related toxicity remained low. Among patients receiving radiotherapy, grade ≥ 3 acute and late toxicities were uncommon, and no grade 4–5 radiotherapy-related adverse events were observed. This favorable safety profile is particularly relevant given the advanced age and substantial comorbidity burden of the study population. These findings are consistent with those reported by Meixner et al., who evaluated women aged ≥ 80 years treated with adjuvant radiotherapy and observed low rates of grade ≥ 3 toxicity, with most patients completing the planned treatment [25]. Similar low morbidity has been reported by Rovirosa et al. and others using contemporary radiotherapy approaches in elderly cohorts [5,31].

From a practical perspective, the most challenging adjuvant radiotherapy decision in very elderly patients may be whether pelvic EBRT is warranted. Vaginal brachytherapy generally requires relatively few treatment sessions, has limited toxicity, and causes little disruption to daily activities. In contrast, conventionally fractionated EBRT requires repeated hospital visits over several weeks and exposes a larger volume of normal tissues, potentially imposing a greater treatment burden in patients with comorbidities, frailty, mobility limitations, or dependence on caregivers. Therefore, in patients aged ≥ 80 years, the expected benefit of EBRT in reducing pelvic recurrence should be weighed against treatment burden and the individual patient’s clinical condition rather than chronological age alone.

Shorter and simplified vaginal brachytherapy schedules may further improve treatment compliance and access in older patients, as suggested by previous studies evaluating hypofractionated regimens with acceptable toxicity and oncologic outcomes [31,32,33]. Such strategies are particularly relevant in this age group, where treatment tolerance, logistics, and quality of life are critical considerations. In this context, the independent association between VB and improved local control, together with the low incidence of severe toxicity observed among radiotherapy-treated patients in our cohort, supports the feasibility of brachytherapy as an adjuvant treatment option in carefully selected very elderly patients.

The association between vaginal brachytherapy and improved overall survival observed in the univariate analysis was attenuated after multivariable adjustment and was no longer statistically significant (aHR 0.71; 95% CI 0.49–1.03; *p* = 0.073). The absence of a statistically significant association between radiotherapy and overall survival despite its association with improved local control may partly reflect the substantial competing risk of non-cancer mortality in this very elderly population. Given the retrospective design of the study, this finding may also be influenced by selection bias, as patients selected for vaginal brachytherapy alone may have had more favorable clinicopathological features and better functional status than those receiving external beam radiotherapy or no adjuvant treatment. Therefore, this association should not be interpreted as evidence of a direct survival benefit attributable to brachytherapy itself but rather as a hypothesis-generating signal.

Our study is not without limitations. Its retrospective nature and inter-institutional heterogeneity in RT indications, techniques, and follow-up protocols may introduce bias. The long study period (2003–2019) may also have introduced temporal heterogeneity related to changes in staging, radiotherapy techniques, and clinical practice. There is also potential for selection bias: the decision to administer radiotherapy and the choice of modality were not randomized and may have been influenced by tumor characteristics, comorbidity, and overall patient status. Additionally, data collection on low-grade toxicities may have been suboptimal or underreported, potentially leading to an underestimation of mild side effects. The absence of formal geriatric assessment using instruments such as the G8 or CGA further limits the evaluation of functional and frailty status.

In addition, competing-risk analyses were not performed. In this very elderly population, death from causes other than endometrial cancer represents an important competing event, particularly for recurrence-related outcomes and cancer-specific survival. Because standard Kaplan–Meier methods may overestimate event rates in the presence of competing risks, these estimates should be interpreted with caution [34].

An additional limitation is the lack of molecular classification, which was not routinely implemented during the study period (2003–2019). Consequently, treatment decisions were based on conventional clinicopathological risk factors, reflecting real-world clinical practice at that time. Future studies integrating molecular classification with clinical and geriatric assessment may further refine prognostic stratification and optimize individualized adjuvant treatment selection in this growing patient population.

Nonetheless, this study includes one of the largest samples of women aged ≥ 80 years with surgically treated endometrial cancer, including a substantial proportion receiving adjuvant radiotherapy, thus offering a valuable complement to the existing literature. Given the aging population and increasing incidence of endometrial cancer, our findings support moving away from chronological age as a sole determinant and toward individualized, risk-adapted approaches. Comprehensive geriatric assessment may contribute to treatment decision-making in multidisciplinary tumor boards. The independent association of VB alone and combined EBRT + VB with improved local control, together with the low incidence of severe toxicity among radiotherapy-treated patients, supports the feasibility of adjuvant radiotherapy in appropriately selected very elderly patients. Treatment selection should therefore be individualized according to tumor-related risk factors, comorbidity burden, and overall patient fitness rather than chronological age alone.

## 5. Conclusions

This multicenter study, representing one of the largest national series of women aged ≥ 80 years with surgically treated endometrial cancer, suggests that adjuvant radiotherapy was associated with favorable local control and a low incidence of severe treatment-related toxicity in carefully selected older patients. In particular, VB alone and combined EBRT + VB were independently associated with better local control.

Our findings support treatment individualization based on tumor characteristics and comorbidity burden rather than chronological age alone. Notably, comorbidity burden, but not age ≥ 85 years, was independently associated with overall survival, emphasizing the importance of considering comorbidity when evaluating very elderly patients for adjuvant treatment. Given the retrospective nature of the study and the absence of molecular classification, these results should be interpreted with caution. Future prospective studies integrating molecular profiling with comprehensive geriatric assessment are warranted to further optimize personalized adjuvant treatment strategies in this growing patient population.

## Figures and Tables

**Table 1:** Baseline characteristics according to radiotherapy administration.

Characteristic	No Radiotherapy	Radiotherapy	*p*
Age			
80–84	76 (60.3%)	174 (76.7%)	
≥85	50 (39.7%)	53 (23.3%)	0.001
Charlson Comorbidity Index			
0–4	37 (29.8%)	48 (21.1%)	
≥5	87 (70.2%)	178 (78.8%)	0.073
Histology subtype			
Endometrioid	84 (66.7%)	158 (69.6%)	
Serous	18 (14.3%)	48 (21.1%)	
Clear cell	7 (5.6%)	9 (4.0%)	
Carcinosarcoma	11 (8.7%)	6 (2.6%)	
Dedifferentiated	1 (0.8%)	1 (0.5%)	
Other	4 (3.2%)	3 (1.3%)	
Unknown	1 (0.8%)	2 (0.9%)	0.110
LVSI			
No	67 (53.2%)	123 (54.2%)	
Yes	30 (23.8%)	83 (36.6%)	
Unknown	29 (23.0%)	21 (9.3%)	0.116
Grade			
1	36 (28.6%)	58 (25.6%)	
2	36 (28.6%)	81 (35.7%)	
3	51 (40.5%)	85 (37.4%)	
Unknown	3 (2.4%)	3 (1.3%)	0.524
FIGO			
I–II	111 (88.1%)	184 (81.1%)	
III–IV	13 (10.3%)	40 (17.6%)	
Unknown	2 (1.6%)	3 (1.3%)	0.183

Abbreviations: LVSI: lymphovascular space invasion; FIGO: International Federation of Gynecology and Obstetrics.

**Table 2:** Univariable and multivariable analysis of prognostic factors associated with overall survival.

	Univariable Analysis			Multivariable Analysis		
	HR	95% CI	*p* Value	aHR	95% CI	*p* Value
Age						
80–84	Reference					
≥85	1.49	1.09–2.05	0.013	1.25	0.87–1.79	0.226
Charlson Index						
0–4	Reference					
≥5	1.52	0.96–2.42	0.075	2.36	1.30–4.26	0.005
Histology		Global * *p* < 0.001				
Endometrioid	Reference					
Serous	2.24	1.56–3.22	<0.001	2.08	1.30–3.33	0.002
Clear cell	2.88	1.48–5.61	0.002	2.04	0.88–4.70	0.094
Carcinosarcoma	3.18	1.29–7.82	0.011	2.71	1.03–7.13	0.043
Dedifferentiated	7.00	5.03–9.75	<0.001	7.52	4.03–14.03	<0.001
Other	1.64	0.41–6.65	0.49	1.88	0.49–7.18	0.357
Tumor Grade		Global *p* < 0.001				
Grade 1	Reference					
Grade 2	1.02	0.70–1.49	0.910	1.11	0.76–1.63	0.576
Grade 3	2.069	1.42–2.98	<0.001	1.60	0.94–2.70	0.080
Type of surgery		Global *p* = 0.227				
HA + DA	Reference					
HA + DA + SLNB	0.75	0.53–1.07	0.117			
HA + DA + LA	1.09	0.67–1.78	0.721			
FIGO stage						
I–II	Reference					
III–IV	1.00	0.99–1.01	0.444			
Lymphovascular space invasion						
Absence	Reference					
Presence	0.99	1.00–1.01	0.672			
Radiotherapy		Global *p* = 0.053				
No radiotherapy	Reference					
EBRT	1.24	0.71–2.17	0.459	1.24	0.69–2.24	0.479
VB	0.63	0.42–0.93	0.021	0.71	0.49–1.03	0.073
EBRT + VB	0.79	0.56–1.13	0.200	0.70	0.47–1.06	0.091

* Global *p* for variables with more than two categories. Abbreviations: CI: confidence interval; HR: hazard ratio; aHR: adjusted hazard ratio; HA: hysterectomy. DA: bilateral salpingo-oophorectomy. SLNB: sentinel lymph node biopsy. LA: lymphadenectomy.

## Data Availability

The original contributions presented in this study are included in the article. Further inquiries can be directed to the corresponding author.

## Supplementary Materials

The following supporting information can be downloaded at: https://www.mdpi.com/article/10.3390/jpm16100520/s1, Table S1: Baseline Covariate Balance Between the Radiotherapy (RT) and Non-Radiotherapy (No RT) Groups Before and After Stabilized IPTW Adjustment; Table S2: Multivariable analysis using conventional Cox regression and applying IPTW weighting.

## Abbreviations

The following abbreviations are used in this manuscript:

RT	Radiotherapy
EBRT	External beam radiotherapy
VB	Vaginal brachytherapy
CTCAE	Common terminology criteria for adverse events
CCI	Charlson Comorbidity Index
ESTRO	European Society for Radiotherapy and Oncology
ESMO	European Society of Medical Oncology
ASTRO	American Society of Radiation Oncology
LVSI	Lymphovascular space invasion
PORTEC-4A	Postoperative Radiation Therapy in Endometrial Carcinoma 4a
RAINBO	Refining Adjuvant treatment in endometrial cancer based on molecular features
FIGO	International Federation of Gynecology and Obstetrics
CT	Computed tomography
MRI	Magnetic resonance imaging
PET/TC	Positron emission tomography
LC	Local control
RC	Regional control
DFS	Disease-free survival
OS	Overall survival
CSS	Cancer-specific survival
VIF	Variance inflation factor
IPTW	Inverse probability of treatment weighting
HR	Hazar radio
aHR	Adjusted hazard ratio
CI	Confidence interval
SMD	Standardized mean difference
HA	Hysterectomy
DA	Bilateral salpingo-oophorectomy
SLNB	Sentinel Lymph node biopsy
LA	Lymphadenectomy
G8	Geriatric 8
CGA	Comprehensive geriatric assessment
