# Supplementary material for: Adjuvant Radiotherapy in the Oldest-Old: A National Spanish Multicenter Analysis of Endometrial Cancer Patients Aged ≥ 80 Years
Source: J Pers Med. 2026 Oct 6;16(10):520. doi: 10.3390/jpm16100520 (PMC13705953; doi:10.3390/jpm16100520)
Supplement: Supplementary file 1 [file jpm-16-00520-s001.zip › jpm-4488097-supplementary.pdf]

Table S1. Baseline Covariate Balance Between the Radiotherapy (RT) and Non-Radiotherapy (No RT) Groups Before and After Stabilized IPTW Adjustment.

**Variable**	**Unadjusted Sample (Weight Off)**	**Initial SMD**	**IPTW Adjusted Sample (Weight By)**	**Final SMD**
**Age*** (80–84 years)	60.3% vs. 76.7%	**0.359**	72.0% vs. 71.9%	**0.002**
**Charlson Comorbility Index*** (Score 0–4)	29.8% vs. 21.2%	**0.198**	23.2% vs. 23.8%	**0.014**
**Histology*** (Endometrioid)	67.2% vs. 69.9%	**0.128**	66.9% vs. 69.0%	**0.091**
**Tumor grade*** (Grade 1)	29.3% vs. 25.9%	**0.147**	26.2% vs. 26.9%	**0.040**
Type surgery (HA +DA)	76.0% vs. 55.8%	**0.436**	74.6% vs. 57.8%	**0.360**
FIGO stage (Stage I)	81.8% vs. 83.0%	**0.032**	81.8% vs. 83.0%	**0.032**
LVSI (Absent/0)	49.6% vs. 54.3%	**0.094**	49.6% vs. 54.3%	**0.094**

***Variables included in the multivariate analysis**

Abbreviations: IPTW: Inverse Probability of Treatment Weighting; SMD: Standardized Mean,Difference; RT: Radiotherapy; FIGO: International Federation of Gynecology and Obstetrics; HA + DA: hysterectomy and double adnexectomy; LVSI: lymphovascular space invasion

Note: Proportions represent the percentage within each treatment group (No RT vs. RT).

An SMD < 0.10 indicates an optimal balance between groups.

Prior to inverse probability of treatment weighting (IPTW) adjustment, substantial imbalances were observed between the study groups, particularly regarding age (80–84 years; SMD = 0.359) and type of surgery (HA + DA; SMD = 0.436).

Following the application of IPTW weights, an optimal balance (defined as a final SMD < 0.10) was successfully achieved for the vast majority of baseline covariates. Specifically, differences in the distribution of age (SMD = 0.002), Charlson Comorbidity Index (SMD = 0.014), tumor grade (SMD = 0.040), and histology (SMD = 0.091) were drastically reduced, ensuring group comparability across these factors. FIGO stage and lymphovascular invasion maintained their initial balanced distribution (SMD = 0.032 and SMD = 0.094, respectively).

However, type of surgery (HA + DA) exhibited a clinically relevant residual imbalance, showing minimal changes in its proportions and maintaining an elevated post-adjustment SMD (SMD = 0.360), this was one of the three variables not included in the multivariate analysis—along with FIGO stage and Linfovascular invasion — due to failing to meet the established statistical significance threshold of p < 0.20 in the univariate analysis.

Table S2. Multivariable analysis using conventional Cox regression and applying IPTW weighting.

Variable	Conventional Cox regression			IPTW Adjusted		
	cHR	95% CI	p value	aHR	95% CI*	p value
**Age**						
80-84 (ref.)						
≥85	1.41	0.95-2.10	0.091	1.25	0.87-1.79	0.226
**Charlson Comorbidity Index**						
0-4 (ref.)	1.56	0.97-2.51	0.066	2.36	1.30-4.26	0.005
≥5						
**Histology**						
Endometrioid (ref.)						
Serous	3.13	1.92-5.10	<0.001	2.08	1.30-3.33	0.002
Clear cell	2.40	1.16-4.98	0.019	2.04	0.88-4.70	0.094
Carcinosarcoma	1.22	0.57-2.60	0.616	2.71	1.03-7.13	0.043
Dedifferentiated	6.87	1.58-29.91	0.010	7.52	4.03-14.03	<0.001
Other	2.37	0.73-7.75	0.153	1.88	0.49-7.18	0.357
**Tumor Grade**						
Grade 1 (ref.)						
Grade 2	1.31	0.78-2.21	0.303	1.11	0.76-1.63	0.576
Grade 3	1.98	1.12-3.48	0.019	1.60	0.94-2.70	0.080
**Radiotherapy**						
No radiotherapy (ref.)						
EBRT	1.52	0.86-2.72	0.153	1.24	0.69-2.24	0.479
VB	0.84	0.52-1.37	0.483	0.71	0.49-1.03	0.073
EBRT + VB	0.091	0.57-1.45	0.685	0.70	0.47-1.06	0.91

*CI for the IPTW model were derived strictly from the bootstrap resampling procedure with 1000 interactions

cHR: conventional HR

aHR: IPTW adjusted HR

EBRT: external beam radiotherapy

VB: vaginal brachytherapy

EBRT+VB: external beam radiotherapy + vaginal brachytherapy
